# Improving the Quality of Maternal and Neonatal Care: the Role of Standard Based Participatory Assessments

**DOI:** 10.1371/journal.pone.0078282

**Published:** 2013-10-22

**Authors:** Giorgio Tamburlini, Klara Yadgarova, Asamidin Kamilov, Alberta Bacci, for the The Maternal and Neonatal Care Quality Improvement Working Group

**Affiliations:** 1 European School for Maternal Neonatal Child and Adolescent Health, Trieste, Italy; 2 Tashkent Institute for Postgraduate Medical Education, Tashkent, Uzbekistan; 3 Ministry of Public Health of the Republic of Uzbekistan, Tashkent, Uzbekistan; Foundation IRCCS Policlinico S. Matteo, Italy

## Abstract

**Background:**

Gaps in quality of care are seriously affecting maternal and neonatal health globally but reports of successful quality improvement cycles implemented at large scale are scanty. We report the results of a nation-wide program to improve quality of maternal and neonatal hospital care in a lower-middle income country focusing on the role played by standard-based participatory assessments.

**Methods:**

Improvements in the quality of maternal and neonatal care following an action-oriented participatory assessment of 19 areas covering the whole continuum from admission to discharge were measured after an average period of 10 months in four busy referral maternity hospitals in Uzbekistan. Information was collected by a multidisciplinary national team with international supervision through visit to hospital services, examination of medical records, direct observation of cases and interviews with staff and mothers. Scores (range 0 to 3) attributed to over 400 items and combined in average scores for each area were compared with the baseline assessment.

**Results:**

Between the first and the second assessment, all four hospitals improved their overall score by an average 0.7 points out of 3 (range 0.4 to 1), i.e. by 22%. The improvements occurred in all main areas of care and were greater in the care of normal labor and delivery (+0.9), monitoring, infection control and mother and baby friendly care (+0.8) the role of the participatory action-oriented approach in determining the observed changes was estimated crucial in 6 out of 19 areas and contributory in other 8. Ongoing implementation of referral system and new classification of neonatal deaths impede the improved process of care to be reflected in current statistics.

**Conclusions:**

Important improvements in the quality of hospital care provided to mothers and newborn babies can be achieved through a standard-based action-oriented and participatory assessment and reassessment process.

## Introduction

Substandard quality of maternal and neonatal care seriously hampers the achievement of desired health outcomes, impedes the fulfilment of women’s and babies’ rights, and causes inefficiency and unjustified direct and indirect costs for the households [1-3]. In spite of this, insufficient attention has been paid to improving the quality of services, particularly in developing and transition countries, where coverage of essential antenatal and perinatal services is high and quality becomes the key limiting factor to achieve results The very recent emphasis on the quality gap has produced a variety of suggested approaches and indicators [4,5], but has not been accompanied by sufficient evidence of what works and how. Indeed, reports of successfully completed quality cycles on the quality of delivery care are very scanty, particularly if we move beyond small scale or narrow-focused interventions [6]. 

We report the results of a nation-wide effort to improve quality of maternal and neonatal hospital care in Uzbekistan, a lower-middle income country with high coverage of antenatal and delivery care, focusing on the role played by standard-based participatory assessments proposed by WHO [2].

## Methods

### Setting

Uzbekistan is a Central Asian country with a population of 29.340.000. Over 97% of births take place in health facilities and 99% are attended by health professionals. The education level is good, with a female secondary school enrolment of women of almost 100% and fertility rate has decreased to less than 3 per thousand [7]. In such a favourable situation, the officially reported data on maternal mortality (28/100.000) and infant mortality (34/1000) [7,8] which are believed to underestimate reality [9] although showing a decreasing trend, are unacceptably high. Neonatal deaths represent over 50% of all child deaths and most of the main causes of death in the neonatal period are asphyxia, prematurity and infections [7,9], a great proportion of which are preventable or treatable [10]. For these reasons, Uzbekistan is among the priority countries included in the UN Global Strategy [11].

Over the last decade, the Ministry of Health of Uzbekistan, in partnership with WHO, UNICEF and the European Commission and with the financial support of the Asian Development Bank, has taken several measures to improve the quality of maternal, newborn and child care, including revision of laws, norms and regulations, in-service training for health professionals in effective perinatal care, development and/or update of clinical guidelines and protocols, implementation of the International Live Birth Definition (ILBD), regionalization of at risk deliveries; renovation of maternity hospitals and provision of essential equipment. Among the efforts to improve quality, starting in early 2010, a participatory and action-oriented quality assessment of hospital care for mothers and newborn babies was carried out in nine out of the 15 perinatal referral centres existing in the country. 

### Assessment tool and methods

The Assessment Tool for the Quality of Hospital care for Mothers and Newborn babies (ATQMN) is aimed at helping hospital managers and health professionals at facility level and policy makers at national level to identify areas of obstetric and neonatal care that need to be improved and to develop plans of action to implement change [12]. The tool is based on WHO guidelines [13-16] and on previous experiences conducted globally for paediatric care [17-19]. All main aspects of care, including infrastructure, equipment and support services, safety and effectiveness of case management and the right to information, privacy, and holistic care for mothers and babies, are covered. Over 400 items are assessed based on four information sources: visit to services and wards, examination of admitted patients and medical records of critical conditions, interviews with staff and mothers. By combining the information from the various sources a score is attributed to each item based on the following criteria: 3= care corresponding to international standards (no need for improvement or need for marginal improvement); 2= substandard care but no serious hazard to health or violation of human rights (need for improvement); 1 = inadequate care with consequent serious health hazards or violation of women’s rights to information, privacy or confidentiality and/or to children’s rights, (need for substantial improvement); 0= very poor care with consequent systematic and severe hazards to the health of mothers and/or newborns, e.g. systematic omission of potentially life-saving interventions or serious gaps for key procedures such as caesarean section, blood transfusion, neonatal resuscitation, etc. (need for thorough revision of structure, organization, procedures and case management related to specific items or to the whole area).The scores for each item are merged in one summary quality of care score for each area so that an overall view of priority areas is obtained. 

The findings of the assessment are discussed with hospital managers and key staff at the end of the assessment process, which normally requires from 2 to 3 days for each hospital . A plan of action is developed to address the main gaps, focusing on those that can be addressed at facility level. Following the assessment of a representative sample of maternity hospitals, the assessors' team reports to the Ministry of Health and to partners about the findings, including a list of actions that need to be taken at health system level (standards, norms and regulations, financing issues, health information issues, human resources management, equipment, drugs and supplies) to address the common systemic gaps. A more detailed description of the tool and of the methods of the assessment has been provided elsewhere [2]. 

The assessment team included 15 national professionals (three hospital managers, three neonatologists, three obstetricians, three midwives, and three interviewers) supervised by an international expert. The reassessment team included six national assessors and the supervisor. The numerosity of the team was justified by the need to build national capacity by training a pool of national experts. Prior to the first assessment, the international expert held a one day workshop, where national trainers were introduced to the tool contents and methods. 

### Selection of sites

The initial assessment was carried out in 2010, according to national plans, in nine referral centers. A sample of four centers was chosen for a short term reassessment, based on geographical distribution across the country and excluding the National Republican Perinatal Center since many national assessors were based there. National assessors did not take part in the assessment or reassessment of centers where they have been working as staff members or as trainers. One of the centers had the first assessment delayed by a few months with respect to the others and was chosen to evaluate to which extent the short time elapsed from the first assessment made a difference.

### Design of the study and data analysis

The study design was a before-and-after observational study. Average scores for each main area of care at first assessment (time 0), and second assessment (time 1) were compared and differences calculated. The following criteria were used to assess the role of the standard based assessment-process in the observed changes a) the presence of a clearly recognizable causal link between the observed change and specific actions included in the action plan as a result of the assessment; b) the actual implementation of the relevant actions in the time elapsed between the two assessments; c) the role that interventions other than the assessment taking place over the same period, such as training, new equipment, modified national regulations etc. could have played in the observed changes. Based on the above criteria, the causal role of the assessment- in the observed changes in quality of care was classified as: absent or marginal; contributory although not exclusive; crucial.

### Ethics approval

The paper reports the results of an observational study assessing the changes occurred in the quality of maternal and neonatal care after an action plan to improve care was developed by the hospital managers and staff of four large perinatal referral centers in Uzbekistan. The activity was planned by the Ministry of Public Health of the Republic of Uzbekistan together with WHO Regional Office for Europe and with the UNICEF and WHO Country Offices, within the EU-funded project aimed at improving the quality of maternal neonatal and child care. UNICEF was the technical agency in charge of coordinating the project in collaboration with the Ministry of Public Health. There was an official request by the Ministry of Public Health to UNICEF on the introduction of the WHO assessment process in Uzbekistan within the project. The assessments methods, including the interviews to staff and patients and the consent procedure, were approved by the Ministry of Public Health of the Republic of Uzbekistan through an official decree. One of the Authors of the paper (AK) was national coordinator, on behalf of MoPH, of the EU/UNICEF project. 

There are no Institutional Review Boards in the hospitals included and all decisions about data collection of whatever kind are taken at central level. However, the hospital directions, based on the above-mentioned decree, approved the assessment and reassessment methods and were actively involved in the study and in the related assessment activities. 

Mothers and staff members were anonymously and confidentially interviewed by members of the national assessment team to assess specific items and to express their opinions. Written consent was not asked to mothers because women would not accept to be involved in an interview if they had to sign papers. The international supervisor, the members of the national assessment team in charge of the interview with mothers and local staff agreed on the fact that the best way to obtain free opinions from women was through an informal approach by people that qualified themselves as health professionals external to the institutions. The interviewers, who were always midwives, clarified the purpose of the interview and of the whole activity to the interviewed mothers, guaranteed the anonymity, and then asked for verbal consent. At least two members of the assessment team were present when mothers were asked for their consent and interviews were done. The interviews, including verbal consent, were not recorded. 

## Results

The reassessment was carried out in April 2011, after an average time of ten months from the previous assessment (12 months in three of the four centers and four months in one centre). Population served by each centre, number of deliveries and of LBW newborn babies, main perinatal indicators, and variation between 2010 and 2011 are shown in Table 1. 

**Table 1:** Main characteristics of hospitals involved in the assessment and reassessment process.

	1	2	3	4
Population served as a referral centre	2 572 800	2 278 400	1 126 400	2 643 500
No. doctors	53	89	41	66
No. nurses and midwives	202	552	193	340
No. deliveries 2009	6613	6721	5052	6532
No. deliveries 2010	7292	7624	5237	6846
Percentage LBW 2009	11,2%	7,7%	6,4%	7,3%
Percentage LBW 2010	12,9%	12,0%	8,3%	8,7%
PNMR 2010 (first trimester)	59,6‰	53,3‰	53,4‰	30,4‰
PNMR 2011 (first trimester)	57,7‰	66,9‰	59,1‰	34,5 ‰
ENMR 2010 (first trimester)	22,8‰	22,8‰	25,6‰	21,3‰
ENMR 2011 (first trimester)	23‰	22,3‰	25,8‰	23,7‰

In the period between the first and second assessment an increase in the number of deliveries, and an even greater increase in the proportion of at risk pregnancies and LBW babies was observed all centers, as a result of the ongoing process of regionalization of at risk cases. As a consequence, an increase in breech presentations, maternal complications and congenital anomalies (detected both prenatally and at birth) was reported in all centers. 

The assessment led to the development of action plans which addressed all aspects of care - such as support services, including statistics and laboratory, basic amenities, case management, and holistic care - that were found deficient and were thought to be manageable at facility level. Table 2a and Table 2b provide a list of the actions planned in the four centers. Plans of action included a time-line and the identification of staff members (hospital managers, heads of departments and services, head nurses/midwives) in charge of each action.

**Table 2:** a. Overview of the action plans developed in the 4 maternity hospitals, by main area of care: support services and obstetric care. b. Overview of the action plans developed in the 4 maternity hospitals, by main area of care: neonatal care, quality procedures and access.

**Table 2a**	
**Main area of care**	**Actions planned (no of hospitals including them out of 4**)
Statistics and records	Periodical audits based on review of main indicators [2]
	Audit of maternal deaths in collaboration with district hospitals [1]
	Improved contents and completeness of medical records [1]
	Introduction of computer -based information system [1]
Drugs and supplies	Improved availability of essential drugs [3]
Equipment	Improved equipment for neonatal care [3]
Laboratory	Improved laboratory equipment and reagents [3]
Infrastructure	Improvement in availability of hot water, toilets, showers [3]
	Improved heating system [1]
Labor ward	Privacy improved [4]
	Adequate heating source for newborn babies [2]
	Overall lay out of labour and delivery room improved [1]
Neonatal ward	Improved equipment in NICU (CPAP, incubators) [3]
Normal delivery	Choice of position in labor offered [4]
	Less use of enemas [3]
	Partogram used for decision making [2]
	Active management of 3rd stage of labor [3]
	Expanded role of Midwives during labour and delivery [3]
	Improved foetal monitoring [2]
	Training of staff in EPC [2]
Caesarean section	Revision of inappropriate indications [2]
	Blood loss assessed [1]
	Antibiotic prophylaxis introduced [1]
Obstetric	Improved use of oxytocin [2]
complications	
	Reduced polypharmacy [3]
	Protocols on correct use of Magnesium sulphate [3]
	Revised protocols for hypertension [1]
	Revised protocols for obstetric complications [1]
	Improved monitoring of preeclampsia [1]
	Training of anesthesiologists in the case management of severe hypertension [1]
**Table 2b**	
**Main area of care**	**Actions planned (no of hospitals including them out of 4**)
Neonatal care	Improved thermal control [3]
	Improved use of Apgar score [1]
	Improved promotion of breastfeeding [1]
	Improved neonatal resuscitation [1]
	Mothers more involved in neonatal care [1]
	Training more staff in effective perinatal care [2]
Neonatal complications	Monitoring charts of newborn babies admitted in NICU filled in [1]
	Calculation of feeding needs [2]
	Mothers involved in care for sick newborn babies [1]
Emergency obstetric care	Privacy improved in obstetric emergency room [1]
	Protocols developed for improved team work [3]
	Protocol for prophylaxis of thromboembolism [1]
Infection control	No vaginal examination in premature rupture of membranes [1]
	Facilities for washing hands improved [4]
	Revised antibiotic prophylaxis protocols [4]
Monitoring and follow up	Improved monitoring of vital signs for sick newborns [3]
	Improved monitoring and recording for mothers [4]
Guidelines	Development of local protocols based on WHO standards [4]
Audit	Audit of maternal deaths introduced [2]
	Audit of near miss and perinatal deaths introduced [1]
Access & referral	Assessment carried out in district hospitals of the catchment area [1]
Mother and baby friendly care	Improved information given to mothers at admission, during hospital stay and at discharge [4]
	Partners/companions allowed in delivery room [3]
	Rooming in after Caesarean Section [2]

Over the time elapsed between the assessments, all four centres received additional inputs, the main ones being new equipment, such as CPAP equipment, oxygen saturimeter for neonatal respiratory support and laboratory equipment for basic biochemistry. Over the previous 5 years, a number of other inputs had been provided by a variety of programs implemented by the MoPH with the support of international agencies, including training in essential neonatal care, emergency obstetrics and essential perinatal care [16], introduction of the BABIES matrix for the analysis of perinatal deaths [20], implementation of ILBD.

Table 3 provides an overview, for all four hospitals, of the changes observed between the first and the second assessment. All hospitals improved their overall score by an average 0.66 points out of 3 (range 0.4 to 1), i.e. by 22%. The hospital that showed the smallest improvement (0.4) was the one reassessed only 4 months after the first assessment. The improvement occurred in all the main areas of care and was greater in case management and particularly in the care of normal labour and delivery (+0.9) in monitoring and infection control and mother and baby friendly care (+0.8), labour ward lay out and obstetric and neonatal complications (+0.7). The observed changes included the adoption of evidence based protocols and internationally recommended practices and the abandonment of inappropriate care practices. For example, in one centre episiotomy went from 26.6% to 0.5% and active management of 3^rd^ stage of labour increased from 0.2 to 85%. A widespread improvement occurred in some basic amenities such as availability of cold and warm water, basic supplies such as soap and antiseptics and in holistic care provided for both mothers and babies. For example, the presence of a companion at delivery went in one centre went up to 88%, and vertical position in labour in another centre increased from 22 to 54%. Statistics were collected regularly, including the use of the BABIES matrix showing outcomes by birth weight and time of death. 

**Table 3:** Change in the average scores for each main area in the four perinatal centres (A,B,C,D) between first [1] and second [2] assessment and estimated role of the Quality Assessment (QA).

	**A 1**	**A 2**	**B 1**	**B 2**	**C 1**	**C 2**	**D 1**	**D 2**	**mean**	**±**	**QA**
Statistics and records	1	1	2	2.6	1	2	2	2.2	1.9	0.4	-
Drugs and supplies	1	2	2	2.5	1	2	2	2.8	2.6	0.8	*
Equipment	2.8	2.8	1.5	3	1	2.5	2	2.8	2.8	1.2	-
Laboratory	0	1	1.5	2	1	2	1	2.4	1.8	0.8	-
Infrastructure	2	2	2	2.5	1	1.2	2.5	3	2.5	0.2	-
Labor ward	2	2.2	1	2.5	2	2.4	2	2.8	2.4	0.7	**
Neonatal ward	3	3	2	2.7	2	2.3	2	2.8	2.7	0.6	*
Normal delivery	1	2	1	2.6	2	2.4	2	2.5	2.4	0.9	**
Caesarean section	2	2	2	2.6	2	2.8	2	2.8	2.5	0.7	*
Obstetric complications	1	1.2	1	2.8	2	2.8	2	2.3	2.2	0.7	*
Neonatal care	2	2	2	2.6	2	2.3	2	2.7	2.4	0.5	**
Neonatal complications	1	2	2	2.7	1.4	1.9	1	1.9	2.1	0.7	*
Emergency obstetric care	1	2	2	2.5	3	3	1.	1.9	2.3	0.5	*
Infection control	1	2	1	2.5	2	2	2	2.7	2.3	0.8	**
Monitoring and follow up	1	1.2	1	2.5	2	2.5	1	2.8	2.2	0.9	*
Guidelines	2	2	1	2.8	2	2	2	2.4	2.3	0.5	**
Audit	2	2	1.5	2.8	2	2.4	2	2.2	2.4	0.5	*
Access to hospital and referral	2	2	2	2.5	1	1.8	2	2.1	2.2	0.3	-
Mother and baby friendly care	2	3	2	2.8	2	2.4	2	2.5	2.7	0.8	**
**Overall**	**1.6**	**2.0**	**1.6**	**2.6**	**1.7**	**2.3**	**1.8**	**2.5**	**2.33**	**0.66**	

Legend: 0=absent or marginal;* contributory;** crucial

An average of 4 to 6 women, including those who had just delivered, those just admitted or those being discharged, were interviewed in each centre. The interviews confirmed progress in areas such as the amount and quality of the information provided, the offer of various options for position during labour, the presence of family members at delivery, etc. Some of the women who were interviewed were multiparae and could compare the current experience of labour and delivery with their previous experiences in the same facility, reporting very positive changes. 

Table 3 also shows the estimated contribution of the assessment and the subsequent action plan for quality improvement. Improvements in most case management areas could be attributed mainly to the assessment, while the progress in the availability of equipment (+1.2), drug supply and laboratory (+0.8), was mainly due to a project supported by the Asian Development Bank. Only marginal improvements were observed in infrastructure and access to hospital, which depend essentially on budgetary allocations and national policies which are beyond the responsibility of the hospital management. 

The reassessment also showed persisting gaps in some areas. For example, although perinatal statistics and mortality indicators were collected more regularly in all 4 centres, the capacity to analyse and interpret and use data them as a guide for action was still insufficient. And although auditing procedures such as near-miss analysis [21] had been introduced, implementation was variable and the ability to learn lessons from case review was still limited. 

Overall, the attitude towards external and internal peer review, assessment, quality improvement appeared to be critically improved with respect to the first assessment, although variability was significant across and within hospitals. In all hospitals, directors and heads of departments acknowledged the role of the assessment process in helping them to identify specific organizational issues, practices, procedures and protocols that needed to be improved and in providing the knowledge background, the references and the motivation for change. Most comments emphasised that the effects of the assessment tool went beyond its quality improvement purposes, since it was used also as a guide to practice, as a promoter of the use of guidelines, as a tool for self assessment, and for internal supervision and coaching. Many staff members reported during reassessment they have been using the tool also for checking specific items or for revising procedures and discussing cases. All professionals emphasized the new, non-judgemental, supportive approach and the fact that using the tool to assess areas that require the collaboration of various teams and services promoted a more collaborative approach and team work among professionals, particularly between obstetric and neonatal teams. 

## Discussion

We were able to document important improvements in the quality of care provided to mother and newborn babies in busy perinatal referral centres of a lower-middle income country over a short period of time. Substantial improvements were observed across a wide range of areas from the availability of basic amenities such as hot water and toilets to holistic care provided to women during labour, delivery, hospital stay and discharge and to improved case management of normal delivery, term and preterm newborn babies. This is, to our knowledge, the first report to document the results of a quality cycle applied to the whole continuum of delivery care from admission to discharge in more than one centre. Similar approaches have been used, and showed results, in the area of paediatric care [19]. 

Some of the observed improvements are attributable to the efforts made by the Government of Uzbekistan and its international partners to upgrade the equipment, ameliorate drug supply, provide training and introduce data management and audit procedures. However, there was close correspondence between the actions included in the action plan developed after the initial quality assessment and most of the observed improvements. Changes were greater in areas that require only basic knowledge and commitment by health professionals, such as the essential care of mothers and babies in normal delivery and infection control, than in the management of complications, which require the acquisition of more complex skills and may require specific technologies. Not surprisingly, improvement was smaller in the centre where the time available to implement the action plan was very short, i.e. only four months. All these circumstances bring support to a causal role of the assessment process in promoting change.

The results were obtained through a participatory process which was able to build awareness of existing gaps and, most important, motivation to change among hospital managers and key staff. The assessment process, besides promoting change within the health facility, prompted action by hospital managers vis-à-vis local and national health authorities to ensure that systemic issues, such as lack of specific commodities, were addressed. Differences across hospitals and within hospitals can be attributed to varying degrees of commitment and leadership. In fact, effective leadership was facilitated by our approach, which provided at the same time an authoritative support for those who wanted to promote change and a comprehensive instrument to guide the process.

Limitations in the assessment methods, particularly regarding independence and comparability of the base-line and follow-up assessments may have flawed our findings and need to be discussed. We made sure to avoid the involvement of national assessors who had been staff members or trainers in the facilities that were to be assessed. The presence of an international consultant as supervisor was a further, although not necessarily absolute, guarantee. Assessors could certainly have been keen to show progress, in recognition of efforts made by hospital management and staff. However, scores were attributed after careful and joint assessment of all items by more than one assessor, which greatly reduced subjectivity in scoring. Comparability and consistency of scoring in baseline and follow-up assessment were ensured by the fact that in all four centres the assessment and reassessment teams coincided almost entirely.

The validity of our findings, which regard improvements in structural and process items, would be obviously strengthened if these were accompanied by improvements in outcomes. In a static and closed system, with a reliable information system, improvements in quality of care in important structural and process areas should be reflected by a decrease in perinatal mortality rate (PMR) and maternal mortality ratio. Unfortunately, several factors impede to show this link. First, the regionalization of perinatal care, a process which was still ongoing at the time of the study, increased the proportion of at risk cases in all centres, as shown by the substantially higher proportion of LBW babies in 2010 with respect to the previous year, and the higher PMR and ENMR. The improved referral would gradually reduce PMR and ENMR in population-based statistics and in national statistics, but not in referral centres. However, population based perinatal statistics are by themselves changing due to improved recording of perinatal deaths and the implementation of the ILBD, which was also still going on at the time of our study. This leads to a temporary increase in neonatal mortality rates, so that even population based statistics do not necessarily reflect the improvement in case management. It should be noted that the implementation of the ILBD contributes much more to the increase in mortality rates then to the proportion of premature/low birth weight cases, since many babies with birth weight between 500 and 1000 gr die, but overall they represent much less than 1% of all births. Thus, if these cases are included in statistics, the effect is marginal on LBW rate but important on mortality rates. Early neonatal mortality rate (ENMR) by weight classes would be a better indicator of improved quality of care than overall ENMR [20] but changes data recording and reliability makes comparison over time and across centres still difficult. With respect to maternal deaths, these are too rare events, at least in Uzbekistan, for changes to achieve significance over a limited period of time and on a limited population, not to mention the fact that they depend also on quality of careseeking and referral [22-24].

Finally, it should be emphasized that improvements in quality of care such as those described are expected to produce, particularly in countries with moderately high mortality rates such as Uzbekistan, mainly in reduction of complications, sequelae and long term disability, which can only be measured with rather sophisticated and expensive ad hoc studies. 

The findings of our work show that a standard based participatory assessment can be an important agent of change through a variety of mechanisms, including: improved knowledge of guidelines and best practices recommended at international level; motivation to change deriving from peer-review through a multiprofessional and supportive approach; detailed action plans developed with clear time-line and responsibilities; and the stimulus provided by the reassessment.

We fully acknowledge the important role of distal determinants such as reproductive health, nutrition, health literacy etc in improving health outcomes [23-26], at least in the medium term, and we recognize that coverage still needs to be improved in many high mortality countries, and for disadvantaged population groups in all countries. However, we believe that more investment needs to be made in quality systems and, within these, in approaches that involve health professionals rather than controlling them [27]. These approaches need to be feasible, well accepted by professionals, and cost-effective [28]. The approach we described compares favourably with other quality improvement methods, which require the creation of a specific bureaucracy at national and local level, are more expensive, and do not promote the peer to peer intra-disciplinary process which is crucial to achieve results based on professional motivation [27,28]. 

Future developments include the establishment of a link between the assessment process and certification/accreditation processes to which health facilities and single departments could voluntarily apply, or could be required to apply, as a basis for performance based bonuses. Since standards, criteria and measurement process have been set and validated, future challenges include building national capacity to run this kind of processes. Based on our experience, this seems to be achievable over a relatively short period of time, provided that a competent and well trained, multidisciplinary group of professionals is available and that national health authorities fully endorse this activity.

### The Maternal and Neonatal Care Quality Improvement Working Group was composed by

Giorgio Tamburlini, Klara Yagdarova, Asamidin Kamilov, Alberta Bacci (coordinators and supervisors) Audrius Mačiulevičius, Neonatologist, Kaunas, Lithuania; Stelian Hodorogea Obstetrician-Gynecologist, Chisinau, Moldova; Irina Stepanova, Midwife, Perm, Russian Federation (international assessors); Rano Aripova, Midwife, Republican Perinatal Centre, Tashkent; Dilorom Abdurakhmanova, Midwife, Perinatal center city of Tashkent; Shakhnoza Sadikova, Midwife, Republican Perinatal Centre (RPC); Yulduz Yuldasheva, Midwife, Republican Specialist Centre for Obstetrics and Gynecology (RSCOG), Axmedova Dilorom, Midwife, Perinatal center city of Tashkent; Nodira Kasimova, Neonatologist, Republican Perinatal Centre; Malika Usmanova, Neonatologist, Perinatal center city of Tashkent; Nodira Irgasheva, Neonatologist, RSCOG; Dilbar Azizova, Neonatologist, Namangan branch of RSCOG; Muattar Razikova, Neonatologist, Tashkent RPC; Kudrat Jumaniyazov, Obstetrician-Gynecologist, Khorezm regional perinatal center, Rustam Yuldashev, Obstetrician-Gynecologist Karshi branch of RSPCOG, Shahida Babadjanova, Obstetrician-Gynecologist, RPC; Natalia Lyubchich, Obstetrician-Gynecologist, RSPCOG; Alexey Klimashkin, Obstetrician-Gynecologist, Lecturer,Tashkent Medical Academy; Nigora Karabaeva, Obstetrician-Gynecologist, Coordinator of Asian Development Bank project; Gultakin Muratova, Obstetrician-Gynecologist, RSPCOG; Nigora Ishmatova, Obstetrician-Gynecologist, Djizak branch of RSPCOG; Feruza Akhrarova, Obstetrician-Gynecologist, *Fergana* regional perinatal center; Fakhriddin Nizamov, National Professional Officer, WHO Country Office, Uzbekistan (national assessors). 
